# The Mediating Role of Coping Style in the Relationship Between Sleep Quality and Burnout: A Cross-Sectional Study Among Psychiatric Nurses

**DOI:** 10.3389/fpsyt.2022.926040

**Published:** 2022-06-23

**Authors:** Ming Yin, Weiqin Li, Qun Yang, Yan Yue, Xiaojia Fang, Zhong Yang, Xinda Wang, Qin Liu, Fanzhen Kong, Caifang Ji, Xiaoli Lv, Hao Wang, Nian Yuan, Zhe Li, Caiyi Zhang, Kan Li, Yang Yang, Xiangdong Du

**Affiliations:** ^1^Suzhou Guangji Hospital, The Affiliated Guangji Hospital of Soochow University, Suzhou, China; ^2^Nanchong Psychosomatic Hospital, Nanchong, China; ^3^Medical College of Soochow University, Suzhou, China; ^4^Affiliated Hospital of Xuzhou Medical University, Xuzhou, China; ^5^The Third People's Hospital of Changshu, Suzhou, China; ^6^Taicang Third People's Hospital, Suzhou, China; ^7^Jiangxi Mental Hospital, Nanchang, China; ^8^Mental Hospital of Yunnan Province, Kunming, China

**Keywords:** nurses, mental health, sleep quality, coping styles, job burnout

## Abstract

**Background:**

Although sleep quality is clearly associated with job burnout as shown in the existing research, the mechanism underpinning such relationship remains undefined. This work, thus, aimed to assess the current situation of sleep quality and burnout in Chinese psychiatric nurses, and to analyze the relationships between sleep quality, burnout and coping style, in order to provide possible targets to enhance mental health and wellbeing among psychiatric nurses.

**Method:**

This cross-sectional study was carried out in seven rehabilitation centers located in four different regions of China. The Pittsburgh Sleep Quality Index, the Epworth Sleeping Scale, the Maslach Burnout Inventory General Survey, and the Coping Style Questionnaire were distributed to 853 nurses in various mental hospitals, with a total of 664 participants being recruited in the final research.

**Results:**

The results of this current study showed a high prevalence of sleep disorders and burnout in Chinese psychiatric nurses. Moreover, emotional exhaustion (*r* = 0.456), cynicism (*r* = 0.323) and negative coping style (*r* = 0.191) in nurses were all positively correlated with total Pittsburgh Sleep Quality Index (PSQI) score, while professional efficacy (*r* = −0.079) and positive coping style (*r* = −0.140) were negatively correlated with total PSQI score. More interestingly, of all negative coping strategies, we found that self-blame had the most significant effect (β = 0.156).

**Conclusions:**

The above results showed that coping style mediates the association of poor sleep quality with job burnout in Chinese psychiatric nurses. This study claimed that there is an urgent need to development the coping skills to sustain a healthy work life for nurses.

## Introduction

Burnout is reflected by physical and mental exhaustion, which refers to prolonged stress response to lasting workplace-related emotional and interpersonal stressors (1). There are three dimensions of burnout, including emotional exhaustion, cynicism and decreased professional performance. Emotional exhaustion is the excessive emotional consumption of individuals, leading to depleted individual resources, which represents the stressful dimension of burnout. Cynicism is a negative, cold or extremely disconnected response to the patient populations served. Decreased performance is an overall sense of one's inefficiency at the workplace, as well as a lack of professional achievement, which represents the dimension of self-evaluation. Compared with other occupations, nursing represents a high-risk of burnout, being admittedly stressful and emotionally demanding (2, 3). The nurse-to-population ratio in China is 1:1,750, far lower than the 1:140 to 1:320 reported in developed countries (4). In the micro level, because of inability to effectively cooperate with the nurse for treatment, suicide risk, self-injury, impulse and violent behavior in mental illness patients, psychiatric nurses are in more risk of serious mental health problems (5–7). On the other hand, the invigorated nursing plays an important role in psychiatric treatment considering the quality of nursing directly affects the mental state of hospitalized psychiatric patients, the degree of treatment cooperation, and even the final outcome (8–10). In this sense, exploring the mediating factors that are possibly related with nurses' job burnout has important implications not only for enhancing the mental health and wellbeing among psychiatric nurses, but also the entire treatment system.

One possible reason for nurses' job burnout can be the poor sleep quality. Nurses cannot be able to get enough sleep because of intense work and frequent shifts. Studies have shown that nurses sleep worse significantly than professionals of other occupations (11, 12). Other reports have identified that psychiatric nurses have poorer sleep even than general hospital nurses (13). Deficient sleep quality can indeed bring with many serious problems, such as irritability, anxiety, fatigue and memory loss (14). These negative emotional issues could decline work enthusiasm, efficiency, and eventually, affecting the nursing performance. Although both sleep problems and job burnout have been paid attention to in the field of public health, the mechanism by which sleep quality affects job burnout in psychiatric nurses has not been explored in previous studies.

Another possible reason is coping style. Coping is the continuous effort of a given individual to change cognition and behavior to cope with demands considered to be stressful and beyond the reach of personal resources (15, 16). Previous evidence suggested that coping styles act a critical mediating role in psychological stress (17). Individual coping style can both affect the properties and strength of response to stress, as well as further regulate the relationship between stress and its results. According to the coping effect, coping style can be divided into positive and negative coping (15, 18). Burnout represents a progressive condition caused by inefficient coping strategies (19). As illustrated in the previous studies, positive coping is negatively associated with and could predict burnout (20, 21). Conversely, negative coping has a positive association with burnout as well (22–25). In addition, coping style, as an individual susceptibility factor, mediates the relationship between stress and sleep quality (26). A report revealed that it is not stress itself that causes sleep disorders, but the evaluation of and response to it (27). Although job burnout and coping style are overtly associated, as well as sleep quality and coping style, the mechanism underpinning such relationships remains undefined this far, which will be investigated directly by this current study.

Admittedly, multiple parameters can affect burnout, but the present work focused on sleep quality and coping style only. This study aimed to (1) understand the current situation of sleep quality and burnout in Chinese psychiatric nurses; (2) evaluate the relation between sleep quality and burnout; and (3) analyze whether coping style mediates the impact of sleep quality on burnout.

## Methods

### Participants

This trial included 853 psychiatric nurses from Jiangsu Province, Jiangxi Province, Sichuan Province, and Yunnan Province in China. The trial was approved by the ethics committee of Suzhou Guangji Hospital. Investigators were psychologists qualified for human studies. The questionnaires were distributed, alongside the explained study aims. Questionnaire filling was anonymous. Individuals agreeing to take part in this trial were required to read the questionnaires and provide answers to all questions at the workplace. The data were collected in Aug. and Sep. 2017.

### Pittsburgh Sleep Quality Index

The Pittsburgh Sleep Quality Index (PSQI) is a self-assessment scale that subjectively evaluates sleep quality in seven areas, including subjective sleep quality, sleep latency, sleep duration, sleep efficiency, sleep abnormalities, hypnotic drug utilization and daily quality of life. Final scores were calculated based on the seven dimensions and it ranged between 0 (best sleep) and 21 (worst sleep), with a score above 7 indicating a sleep abnormality (Cronbach's alpha was 0.83) (28).

### The Epworth Sleeping Scale

The Epworth Sleeping Scale (ESS) subjectively assesses daytime sleepiness, based on eight items (29). The participants are required to fill in a 4-point Likert scale from 0 (never) to 3 (elevated odds) for self-assessment of the odds of “dozing off or falling asleep” in eight situations. Overall scores ranged between 0 (no sleepiness) and 24 (constant sleepiness). Scores of 0–4, 5–10, 11–16, and >16 reflected no, mild, moderate and severe sleepiness, respectively. Internal consistency among these 8 items is high (Cronbach's of 0.73–0.88) (30).

### Maslach Burnout Inventory General Survey (MBI-GS)

The MBI-GS constitutes a 15-item self-reported assessment tool for job burnout, including three dimensions (emotional exhaustion, cynicism and professional efficacy) (1). A Likert scale from 0 (never) to 6 (daily) was utilized for scoring. The broadly utilized Chinese version of the MBI-GS has satisfactory reliability and validity (31). Here, Cronbach's α values in the three dimensions were 0.896, 0.747, and 0.825, respectively.

### Coping Style Questionnaire

The Coping Style Questionnaire has been applied to measure coping style in some existing Chinese studies to assess psychological health of participants. The questionnaire was developed on the basis of Folkman's interactive theory (32), and was adapted from the COPE Scale (33), and the Ways of Coping Questionnaire (34). It aims to evaluate the manner by which a participant dealt with a very stressful event/situation in the last month. Positive coping style, negative coping style and rationalization were measured with 62 items on a 4-point scale (0, never; 1, seldom; 2, sometimes; 3, often). The sub-subscales had a strong reliability of 0.70.

### Statistical Analysis

All data were expressed as mean ± SD. Independent samples *t*-test was carried out for comparisons. Statistical significance was reflected by *P* < 0.05. Pearson correlation was carried out to assess the associations of sleep quality, burnout and coping style. The Baron and Kenny's method was applied for assessing the impact of coping style on the association of sleep quality with burnout (35). SPSS 20.0 was utilized for data analysis.

## Results

### Demographic and Occupational Characteristics

By conducting the initial analysis, 189 questionnaires were excluded due to the missing data, and the final sample consisted of 664 psychiatric nurses, including 138 men (20.78%) and 526 women (79.22%). A total of 210 (31.60%) participants were aged below 25 years, 254 (38.30%) were 26~31 years old, 182 (27.4%) were 32~46 years old, and 18 (2.70%) were 47~52 years old. In relation to marital status, 454 (68.40%) were married, 194 (29.20%) were unmarried, and 16 (2.40%) were divorced. Regarding the level of education, four (0.60%) had only completed lower vocational or lower secondary education, 216 (17.50%) had completed intermediate vocational or intermediate/higher secondary education, and 544 (81.90%) had a college degree or above. With respect to job title, 156 (23.50%) were junior nurses, 241 (36.30%) were intermediate nurses, 210 (31.60%) were senior nurses, and 57 (8.60%) were head nurses. The participants' years of working experience ranged from 0 to 43. In the past one month, 427 nurses (64.30%) had night shifts and 237 (35.70%) had no night shifts.

### Sleep Quality of Nurses

The average PSQI score were 6.87 ± 3.59, i.e., statistically significant difference (*P* < 0.05) compared with the Chinese norm (3.88 ± 2.52). A total of 411 (61.90%) nurses reported that they had good sleep quality, while 255 (38.10%) had poor ones. The total ESS scores in the 664 participants were 9.86 ± 5.07, which also showed statistical significance compared with Chinese control values (*P* < 0.05). There were 107 (16.10%) nurses with normal sleep, 262 (33.39%) with mild sleepiness, 198 (29.82%) with moderate sleepiness, and 97 (14.61%) with severe sleepiness. Total ESS scores in nurses with night shifts in the last month were significantly higher than those of counterparts without night shifts (*t* = 2.90, *P* = 0.004). Similarly, total PSQI scores in nurses with night shifts in the last month were significantly higher than those of individuals without night shifts (*t* = 3.31, *P* = 0.001). Total ESS scores significantly differed by marital status (*F* = 5.60, *P* = 0.004). As shown in the least significant difference (LSD) test, married nurses had the highest ESS scores.

### Job Burnout and Coping Style of Nurses

In this study, the total burnout scores of the 664 participants were 1.96 ± 1.01. Taking the average score of 4 (7-point Likert scale), 265 nurses had a high level of burnout. Job burnout significantly differed by age (*F* = 5.33, *P* = 0.001). After post-hoc LSD test, the burnout scores of nurses aged 32 to 46 and 26 to 31 were significantly higher than those of counterparts under 25 (*P* = 0.000, *P* = 0.007 by LSD test). Total burnout scores also significantly differed by marital status (*F* = 3.78, *P* = 0.023). The burnout scores of divorced nurses were significantly higher than those of unmarried and married colleagues (*P* = 0.009, *P* = 0.029 by LSD test). The level of job burnout was significantly different based on job title (*F* = 2.68, *P* = 0.046). The burnout scores of intermediate and senior nurses were significantly higher than those of junior nurses (*P* = 0.024, *P* = 0.047 by LSD test). Nurses' coping styles for burnout ranged from high to low in frequency: problem-solving, help-seeking, escape-avoidance, rationalization, wishful thinking, and self-blame. There was a significant difference in positive coping style based on age (*F* = 4.83, *P* = 0.002). Nurses younger than 25 years had higher odds of utilizing positive coping style than the other age groups (*P* = 0.004, *P* = 0.000, *P* = 0.030 by LSD test). The negative coping styles were significantly different based on marital status (*F* = 3.20, *P* = 0.042). Divorced nurses used significantly more negative coping styles than unmarried and married colleagues (*P* = 0.016, *P* = 0.044 by LSD test). The use of positive coping styles significantly differed by marital status (*F* = 6.15, *P* = 0.002). Positive coping style scores in unmarried nurses were significantly elevated compared with those of divorced and married nurses (*P* = 0.009, *P* = 0.004 by LSD test). The use of positive coping styles significantly differed by job title *(F* = 6.84, *P* = 0.000). Positive coping style scores in junior nurses were significantly elevated in comparison with those of intermediate and senior nurses (*P* = 0.002, *P* = 0.000 by LSD test).

### Associations of Sleep Quality, Coping Style and Burnout

Table 1 summarizes the results from Pearson correlation analysis. Emotional exhaustion, cynicism, job burnout and negative coping style had significant positive relationships with total ESS score (*P* < 0.01). Emotional exhaustion, cynicism, job burnout and negative coping style had significant positive associations with total PSQI score (*P* < 0.01). Professional efficacy and positive coping style had significant negative relationships with total PSQI score (*P* < 0.01). Job burnout had a significant positive correlation with negative coping style (*P* < 0.01) and a significant positive correlation with negative coping style (*P* < 0.01).

**Table 1:** Means, standard deviations and zero-order correlations (Pearson r) among various parameters.

**Variables**	**Mean**	**SD**	**1**	**2**	**3**	**4**	**5**	**6**	**7**	**8**
ESS	9.86	5.07	1							
PSQI	6.87	3.59	0.286^**^	1						
Emotional exhaustion	1.77	1.20	0.268^**^	0.456^**^	1					
Cynicism	1.37	1.24	0.255^**^	0.323^**^	0.763^**^	1				
Professional efficacy	3.47	1.58	0.019	−0.079^*^	−0.126^**^	−0.217^**^	1			
Job burnout	1.97	1.01	0.178^**^	0.335^**^	0.724^**^	0.764^**^	−0.745^**^	1		
Negative coping styles	0.35	0.19	0.116^**^	0.191^**^	0.275^**^	0.278^**^	−0.177^**^	0.310^**^	1	
Positive coping styles	0.71	0.18	−0.056	−0.140^**^	−0.323^**^	−0.389^**^	0.212^**^	−0.387^**^	0.116^**^	1

* *P < 0.05*.

** *P < 0.01 (two-tailed)*.

### Factors Associated With Job Burnout Amongst Nurses

Job burnout amongst Chinese psychiatric nurses were evaluated by additional dummy-variable regression analyses as presented in Table 2. The dependent variable was job burnout, and independent variables were demographic indexes for which significant differences were found in group comparison analyses, as well as the total ESS score, the total PSQI score, and coping style. At the model 1 level, the coefficient of determination *R*^2^ was 0.02. We next included age, marital status and job title as control variables in the regression analysis, and the adjusted *R*^2^ was 0.34. The results showed that total ESS score, total PSQI score, and escape-avoidance were significant positive predictors of job burnout. Problem-solving constituted a significant negative predictive factor of job burnout, accounting for 34% of its variance.

**Table 2:** Multivariable linear regression analysis of sleep quality, coping style, and job burnout in Chinese psychiatric nurses.

**Variables**	**Job burnout**	
	**Model 1**	**Model 2**
	**β**	**β**
**Independent variables**		
≤25	-0.126	−0.086
32~46	0.102	0.097
47~52	0.001	0.016^*^
Unmarried	0.031	0.056
Divorced	0.074	0.037
Intermediate nurses	0.009	−0.010
Senior nurses	-0.050	−0.108
Head nurses	-0.068	−0.021
ESS		0.054^***^
PSQI		0.200^**^
Self-blame		0.156
Wishful thinking		−0.016
Escape-avoidance		0.168^**^
Rationalization		0.071
Problem-solving		−0.349^***^
Help-seeking		−0.078
*R* ^2^	0.04	0.36
Δ*R*^2^	0.02	0.34
Δ*F*		22.67^***^

* *p < 0.05*,

** *p < 0.01*,

*** *p < 0.001*.

### Mediating Effects of Coping Style on Sleep Quality and Burnout

**Figure 1:**
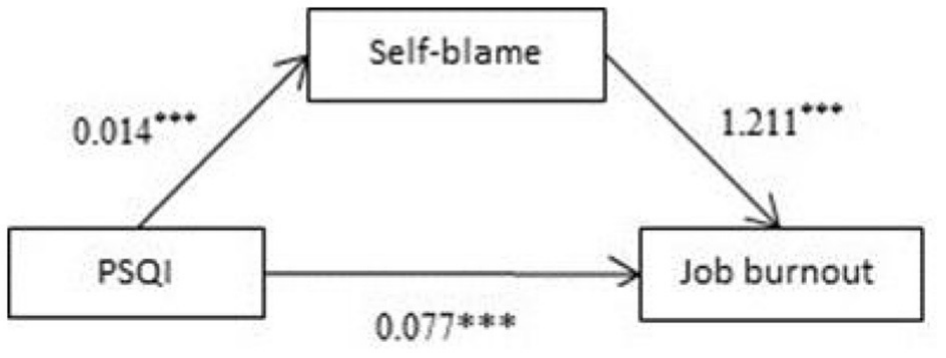
Mediating effects of self-blame on sleep quality and burnout. ***p < 0.001.

Analysis was performed with the Process software (PROCESS-Model#4) (36). The results showed that total PSQI score represented a significant positive predictive factor of self-blame (β = 0.014, *P* < 0.001; Figure 1). Meanwhile, self-blame constituted a significant positive predictive factor of job burnout (β = 1.211, *P* < 0.001; Figure 1). The 95% confidence intervals for the mean did not include zero; therefore, there was a significant mediating effect of self-blame on job burnout (Table 3).

**Table 3:** Mediating effect analysis.

	**Direct effect**	**Standard error**	***T***	***P-*Value**	***LLCI***	***ULCI***
Direct effect	0.095	0.010	9.145	0.000	0.074	0.115

### Mediating Effects of Wishful Thinking on Sleep Quality and Burnout

**Figure 2:**
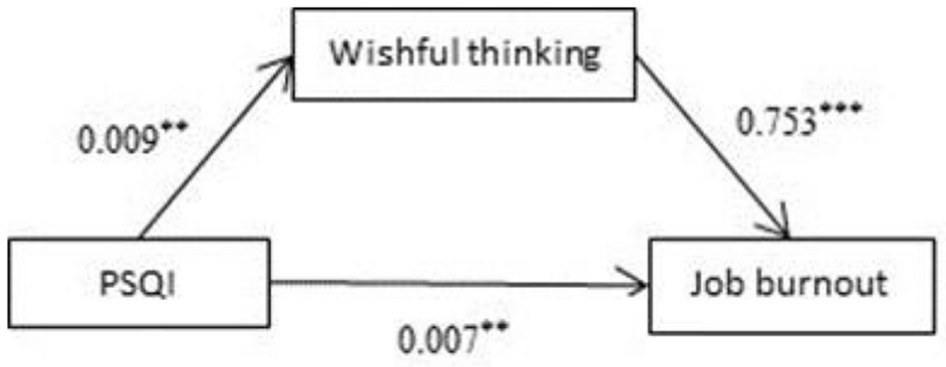
Mediating effects of wishful thinking on sleep quality and burnout. **p < 0.01, ***p < 0.001.

Analysis was performed with Process (PROCESS-Model#4) (36). The results showed that total PSQI score constituted a significant positive predictive factor of wishful thinking (β = 0.009, *P* < 0.01; Figure 2). Meanwhile, wishful thinking was a significant positive predictive factor of job burnout (β = 0.753, *P* < 0.001; Figure 2). The 95% confidence intervals for the mean did not include zero; therefore, there was a significant mediating effect of wishful thinking on job burnout.

### Mediating Effects of Escape-Avoidance on Sleep Quality and Burnout

**Figure 3:**
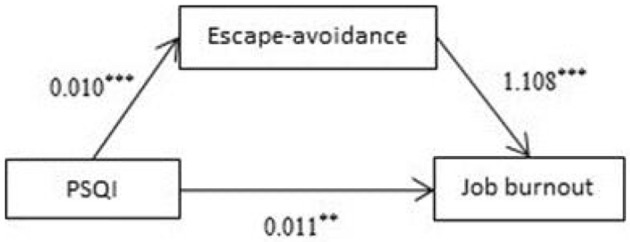
Mediating effects of escape-avoidance on sleep quality and burnout. **p < 0.01, ***p < 0.001.

Analysis was performed with Process (PROCESS-Model#4) (36). The results showed that total PSQI score was a significant positive predictive factor of escape-avoidance (β = 0.010, *P* < 0.001; Figure 3). Meanwhile, escape-avoidance was a significant positive predictive factor of job burnout (β = 1.108, *P* < 0.001; Figure 3). The 95% confidence intervals for the mean did not include zero; therefore, there was a significant mediating effect of escape-avoidance on job burnout.

### Mediating Effects of Rationalization on Sleep Quality and Burnout

**Figure 4:**
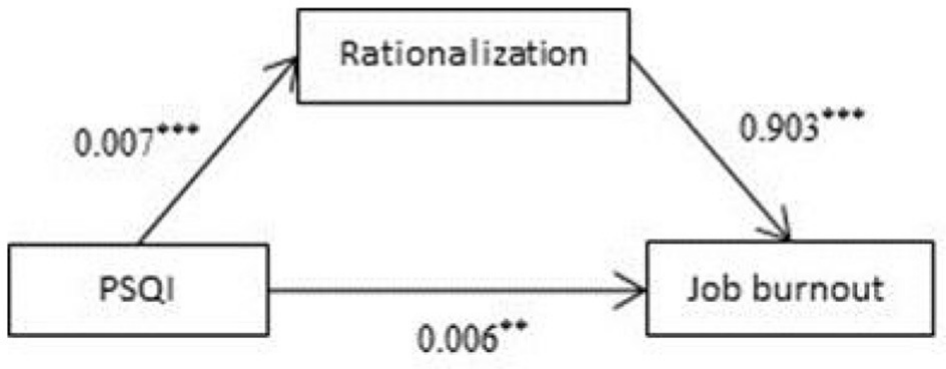
Mediating effects of rationalization on sleep quality and burnout. **p < 0.01, ***p < 0.001.

Analysis was carried out with Process (PROCESS-Model#4) (36). The results showed that total PSQI score was a significant positive predictive factor of rationalization (β = 0.007, *P* < 0.001; Figure 4). Meanwhile, rationalization was a significant positive predictive factor of job burnout (β = 0.903, *P* < 0.001; Figure 4). The 95% confidence intervals for the mean did not include zero; therefore, there was a significant mediating effect of rationalization on job burnout.

### Mediating Effects of Problem-Solving on Sleep Quality and Burnout

**Figure 5:**
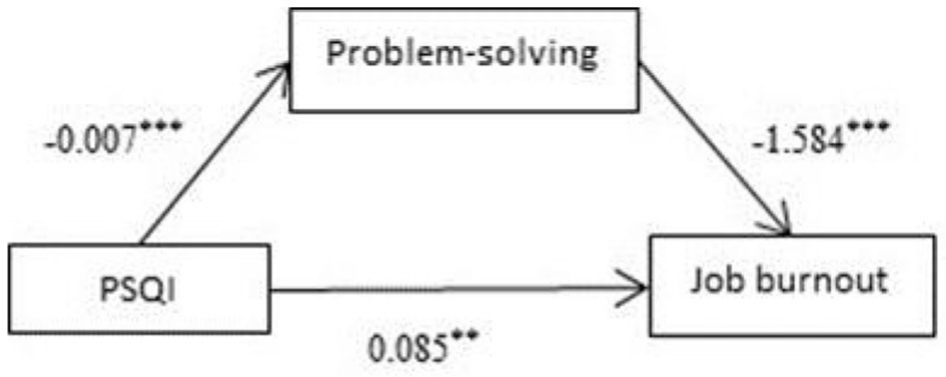
Mediating effects of problem-solving on sleep quality and burnout. **p < 0.01, ***p < 0.001.

Analysis was performed with Process (PROCESS-Model#4) (36). The results showed that total PSQI score was a significant negative predictive factor of problem-solving (β = −0.007, *P* < 0.001; Figure 5). Meanwhile, problem-solving was a significant negative predictive factor of job burnout (β = −1.584, *P* < 0.001; Figure 5). The 95% confidence intervals for the mean did not include zero; therefore, there was a significant mediating effect of problem-solving on job burnout.

### Mediating Effects of Help-Seeking on Sleep Quality and Burnout

**Figure 6:**
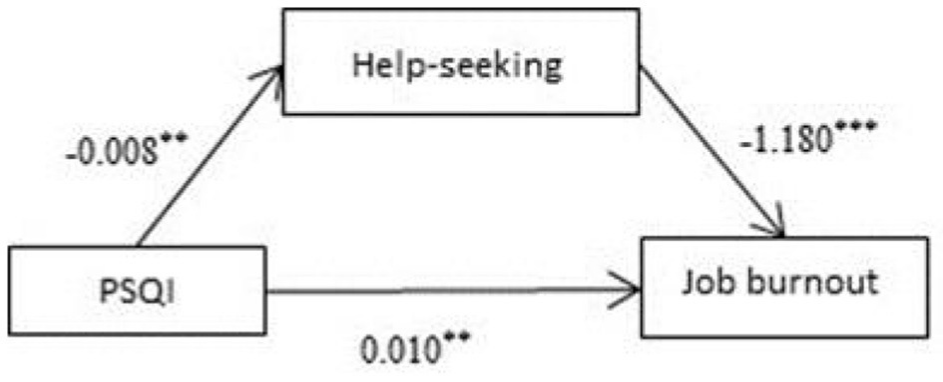
Mediating effects of help-seeking on sleep quality and burnout. **p < 0.01, ***p < 0.001.

Analysis was performed with Process (PROCESS-Model#4) (36). The results showed that total PSQI score was a significant negative predictive factor of help-seeking (β = −0.008, *P* < 0.01; Figure 6). Meanwhile, help-seeking was a significant negative predictive factor of job burnout (β = −1.180, *P* < 0.001; Figure 6). The 95% confidence intervals for the mean did not include zero; therefore, there was a significant mediating effect of help-seeking on job burnout.

## Discussion

The results of this current study showed a high prevalence of sleep disorders and burnout in Chinese psychiatric nurses. Moreover, the present trial assessed the associations of sleep quality, burnout and coping style in Chinese psychiatric nurses, revealing that coping style might have both direct and indirect effects on burnout. In addition to sleep promotion, improving coping skills as an approach to ameliorate mental health in nurses is critical. Therefore, coping strategies should be developed for burnout prevention in nurses.

The results of this survey showed a high prevalence of sleep disorders in Chinese psychiatric nurses, which was higher than that of general nurses shown in the previous research (37). This suggested that the sleep quality of psychiatric nurses is more seriously altered than that of general nurses due to their special occupation, with night shift psychiatric nurses having more problems with sleep quality and sleepiness in particular as further shown in this study. In addition to the working patterns, the problem was also sensitive to marriage status that married nurses reported a worse sleeping. This survey generally showed that 39.9% of nurses' job burnout was at a high level, and this figure is larger than the 34% reported by Aiken LH in American (38). One possible reason for this difference is that psychiatric nurses in China are applying support for more severe mental illnesses. Among the three sub-domains of job burnout, low professional efficacy is one of the most serious problems. This may be due to the psychiatric nurses need to deal with patients who do not cooperate with treatment. It appears that older and higher professional title nurses experienced increased burnout compared with younger counterparts, which is inconsistent with previous studies (37, 39). This may be explained by the fact that older, higher professional title nurses take on heavy responsibilities, undertake heavier nursing workload, face critical patients and emergencies, and supervise nurses with less seniority, which costs more energy. Meanwhile, they might face much more complex pressures from family and daily life, including marriage, child care and education, lacking free time for leisure and relaxation. This can also explain the results that that young, unmarried and low-professional title nurses used more positive coping methods, as they are less likely being challenged by the lower family and financial issues. Interestingly, divorced nurses preferred to use negative coping styles and experienced elevated burnout. Based on these descriptive findings, hospital administrators should be attentive to the burnout problems of psychiatric nurses, and particularly prioritizing the older, divorced, and high-job title nurses, in order to help them cultivate positive coping strategies.

According to Folkman and Lazarus's model of cognitive interaction, coping style can be divided into positive coping (problem-solving and/or help-seeking), negative coping (self-blame, wishful thinking and/or escape-avoidance), and rationalization (15). In this study, we found that emotional exhaustion, cynicism and negative coping style in nurses were positively correlated with total ESS and PSQI scores. Meanwhile, both the professional efficacy and positive coping style had significant negative correlation with total PSQI score. This is consistent with previous studies. Early studies assessing the association of stress with sleep quality indicated that stressful life events could predict sleep disorders in individuals. However, recent studies showed that the cause of sleep disorders is not stress itself, but the evaluation of and coping with stress (40, 41). Positive coping makes people more skilled in coping with pressure in life and work, which can reduce stress response in patients, and thus improve the quality of sleep (42). Negative coping people cannot deal with stress correctly, and tend to indulge in stressful situations and not be resolved, aggravating stress, thereby reducing their sleep quality (43). The results in this study further support coping style represented a potential predictive factor of job burnout in earlier studies (31, 44). These data suggested that negative and positive coping aggravates and alleviates burnout in nurses, respectively.

Sleep disturbance is an important risk factor for emotional and physical exhaustion (45). On the other hand, sleep disorders are also the most common symptoms of job burnout. We demonstrated that coping style had a mediating impact on the association of sleep quality with burnout in nurses. Sleep problems may elicit coping patterns, which in turn cause job burnout. In addition to dealing with sleep problems, improving coping skills is critical to reducing burnout among nurses. Studies by Spataro et al. (46) have shown that an increase in self-blame coping style can lead to job burnout in female medicine residents (47). In consistent with the predecessor's research, of all negative coping strategies, we found that self-blame had the most significant effect. The results showed that total PSQI score represented a significant positive predictive factor of self-blame, which in turn significantly predicted job burnout. Overgeneralized self-blame, which leads to uselessness and hopelessness, is a core symptom of major depressive disorder and a predictor of relapse (48). Increased utilization of self-blame as a coping mechanism could remarkably exacerbate burnout and sleep problems in psychiatric nurses. This suggested that observation of self-blame among nurses may help impede burnout. On the other hand, Chinese culture is considered more collectivist than individualist, and therefore might use a more self-blame coping style (49). Training programs should work toward raising self-compassion and decreasing self-blame (50), especially in nurses with poor sleep quality. More interestingly, of all positive coping strategies, we found that problem-solving had the most significant effect. Prior studies have shown that problem-solving coping style was associated with less sickness absence among female nurses working in hospital care (51). Our study further supports this view. Training programs should work toward raising acknowledging various thoughts concerning the problem, undertaking efforts to understand the situation, predicting the course of events, choosing the most appropriate solutions, planning to solve the problem and implementing this plan as well as taking consistent action to solve the problem.

### Limitations and Strengths

This work had many strengths, including its large sample and a high response rate. We selected hospitals in different regions of China to make the sample representative, and different regions where the hospitals are located reflect different levels of economic development in China. The present findings call for increasing nurses' competency and designing active interventional methods. However, the results and findings reported in this study shall be interpreted with caution as well. Firstly, this research was designed as a cross-sectional trial, from which the causal relationships could not be definitely inferred. In this sense, further longitudinal studies are warranted to further examine these initial findings. Secondly, because all the participants were recruited only from seven rehabilitation centers located in four different regions, these findings have limited generalizability and are difficult to be extrapolated to all the Chinese nurses. At the same time, the sample size was not sufficiently large to analyze possible differences across the regions. This calls for the following researchers who are interested in the nationwide trials in this field. Thirdly, considering this current study only concerned the sleep quality and coping style, some other possible mediating factors, such as mood (e.g., depression and anxiety (52)) can be explored in the future studies. Fourthly, the findings of this research can be deconstructed, for example, specific workplace and individual stressors could be assessed with regard to coping and emotion regulation approaches. Finally, burnout should be examined across multiple facilities and work shifts to provide further data describing the impacts of contextual variables.

## Implications

Poor sleep and mental health in nurses might substantially affect their overall health as well as those of patients. Consequently, nurses should be provided adequate sleep knowledge and information regarding the association of sleep with mental health. We suggest that nursing education should include opportunities dealing with healthy sleep, coping approaches, and mental health promotion, to prevent burnout in nurses. The training about the etiology and signs/symptoms of burnout may help nursing students detect symptoms in themselves and timely seek help. The accurate analysis and determination of a person's coping strategy can help identify the effectiveness of the coping strategy used and provide guidance for studying coping skills and developing therapeutic interventions. To change their PSQI scores, hospitals might provide training nurses on problem-solving skills, which could help develop and apply positive coping styles to mitigate workplace stress (53). Such training should comprise approaches for managing negative affectivity and decreasing disconnected response, e.g., by cognitive reframing (54) and mindfulness-related methods (55, 56), as well as organizational actions addressing burnout by increasing job resources (57). This would ameliorate health and wellbeing in nurses, reducing professional burnout and improving healthcare.

## Relevance Statement

Poor sleep and mental health in nurses might substantially affect their overall health as well as those of patients. High prevalence rates of sleep disorders and burnout were found in Chinese psychiatric nurses. This cross-sectional study demonstrated the mediating effects of coping style on the association of poor sleep quality with burnout symptoms in Chinese psychiatric nurses. Specifically, self-blame coping plays a critical mediating role in this association. In addition to sleep promotion, improving coping skills as an approach to ameliorate mental health in nurses is critical. It is therefore important to improve coping skills to sustain a healthy work life for nurses.

## Data Availability Statement

The raw data supporting the conclusions of this article will be made available by the authors, without undue reservation.

## Ethics Statement

The studies involving human participants were reviewed and approved by Ethics Committee of Suzhou Guangji Hospital. The patients/participants provided their written informed consent to participate in this study.

## Author Contributions

XD: study conception and design, WL, QY, XF, ZY, XW, QL, FK, CJ, XL, HW, NY, ZL, YYu, CZ, KL, YYa, MY, and XD: experiments, MY: data analysis and manuscript writing. All authors contributed to the article and approved the submitted version.

## Funding

This work funded by High-level Health Talents “Six-one Projects” in Jiangsu Province (LGY2020042), “333 Project” Scientific Research Project in Jiangsu Province (BRA2020120), The Suzhou Clinical Medical Center for Mood Disorders (Szlcyxzx202109), Introduction Project of Suzhou Clinical Expert Team (SZYJTD201715), and Scientific and Technological Program of Suzhou (SYS2019112).

## Conflict of Interest

The authors declare that the research was conducted in the absence of any commercial or financial relationships that could be construed as a potential conflict of interest.

## Publisher's Note

All claims expressed in this article are solely those of the authors and do not necessarily represent those of their affiliated organizations, or those of the publisher, the editors and the reviewers. Any product that may be evaluated in this article, or claim that may be made by its manufacturer, is not guaranteed or endorsed by the publisher.
